# Clinical Features and Outcomes of Invasive *Fusarium* Infections from a Singaporean Centre: Is It Time to Redefine How We Assess Treatment Outcomes?

**DOI:** 10.3390/jof11100699

**Published:** 2025-09-26

**Authors:** Yvonne Fu Zi Chan, Benjamin Pei Zhi Cherng, Cherie Le Si Gan, Siew Yee Thien, Sophie Seine Xuan Tan, Hei Man Wong, Yen Ee Tan, Ai Ling Tan, Shimin Jasmine Chung

**Affiliations:** 1 Department of Infectious Diseases, Singapore General Hospital, Singapore 169608, Singapore; benjamin.cherng.p.z@singhealth.com.sg (B.P.Z.C.); cherie.gan.l.s@singhealth.com.sg (C.L.S.G.); thien.siew.yee@singhealth.com.sg (S.Y.T.); sophie.tan.s.x@singhealth.com.sg (S.S.X.T.); wong.hei.man@singhealth.com.sg (H.M.W.); jasmine.chung.s.m@singhealth.com.sg (S.J.C.); 2 Department of Microbiology, Singapore General Hospital, Singapore 169608, Singapore; tan.yen.ee@singhealth.com.sg (Y.E.T.); tan.ai.ling@singhealth.com.sg (A.L.T.); 3 SingHealth Duke-NUS Transplant Centre, Singapore 169608, Singapore

**Keywords:** invasive fusariosis, *Fusarium*, invasive fungal infections, anti-fungal treatment

## Abstract

Invasive *Fusarium* infections pose a significant threat to immunocompromised patients and are characterised by high mortality rates. In this study, we examined 22 unique episodes of proven and probable *Fusarium* infections over a 14-year period at a tertiary hospital in Singapore. Cases were analysed from clinical, microbiological, and radiological perspectives. The most common risk factor for invasive *Fusarium* infections was hematologic malignancy. Fifty percent of patients achieved resolution of infection and were alive at the end of treatment. Conversely, the overall mortality was 50%, with 90% of deaths occurring within three months of the diagnosis of invasive fusariosis; associated risk factors include neutropenia, disseminated infection, and corticosteroid use. Although these deaths would be classified as treatment failures by established criteria; many (8/10; 80%) were due to causes not directly related to invasive fusariosis; such as progression of the underlying malignancy or another infection. We believe that it may be time to redefine how we assess treatment outcomes for invasive mould infections. Nevertheless; invasive fusariosis remains a formidable foe in the immunocompromised host. Early; aggressive treatment with appropriate adjunctive therapies; such as surgery; is crucial for controlling the infection and achieving the best outcomes.

## 1. Introduction

*Fusarium* species are ubiquitous in the environment and are commonly found in soil, water, and air. More than 20 species complexes have been described, with *Fusarium solani* species complex (FSSC) accounting for approximately 50% of cases of invasive infections in humans [1]. The manifestations of infection with *Fusarium* species are diverse and depend on the patient’s immune status. In immunocompetent patients, superficial infections predominate, with onychomycosis, localised skin and soft tissue infections and keratitis being common manifestations [2]. In severely immunocompromised patients such as those with haematological malignancies (HM) and in allogeneic stem cell transplant (ASCT) recipients, *Fusarium* species can cause invasive and life-threatening infections, which are often fatal [1]. Other specific groups of patients in whom *Fusarium* infections have been reported include those with burns [3] and patients admitted to the intensive care unit [4].

The epidemiology, risk factors and treatment response in patients with invasive fusariosis have been detailed in multiple case series [5,6,7,8,9,10,11,12,13,14,15,16,17]. Fusariosis frequently affects patients who are immunocompromised (such as patients with HM, ASCT recipients, or those on immunosuppressants) [14]. It is well reported globally, and in Brazil, two single centre studies found that invasive fusariosis was the second most common invasive fungal infection, comprising approximately 20% of invasive fungal infections [15,16]. Reports from Asia include cases from South Korea, with 14 cases of invasive fusariosis identified from 2003 to 2017, where HM, solid organ transplantation and immunosuppressive therapy were the predominant underlying conditions [10]. Additional reports from Asia include two case series from India. In the series from New Delhi in North India, 10 cases were included, all of which had an underlying HM and/or associated immunosuppressive therapy. The mortality in that case series was 60% and all patients with HM demised [11]. In the other case series from Hyderabad, India, invasive fusariosis occurred in a large proportion of immunocompetent patients who were involved in road traffic accidents. In such cases, affected were treated with surgery with or without anti-fungals and discharged stable [17].

For diagnosis, traditional methods include direct examination of tissue, which may reveal irregular hyaline septate branched hyphae, and a distinct characteristic of the fungus is the production of hyaline banana-shaped macroconidia [1] However, traditional methods are limited by slow turnaround times and low sensitivity. Recent advances in diagnostics include the use of molecular techniques such as multilocus sequence typing, high-resolution melting curve analysis, pan-fungal real-time PCR and next-generation sequencing sequencing [11,18,19,20]. These methods enable rapid and accurate identification to the species level, facilitating quick therapeutic decisions which can lead to improved patient outcomes [1]. However, they are not readily available and may not impact care in resource limited settings.

Treatment is challenging, with limited antifungal options [21] and no established correlation between minimum-inhibitory concentrations (MICs) and outcomes [22,23]. To date, there are no randomized controlled trials in the treatment of invasive fusariosis and treatment recommendations are based on retrospective analyses and expert consensus, reflecting the rarity and severity of the disease. Voriconazole and lipid formulations of amphotericin B remain the mainstay of treatment for invasive fusariosis, in keeping with published guidelines [24,25]. Combination therapy may be the preferred initial treatment approach due to the high MICs of voriconazole [25]. Newer antifungal agents such as fosmanogepix have demonstrated efficacy in the treatment of fusariosis and represents a promising advance in the treatment armamentarium [26].

Invasive fusariosis has a poor prognosis, with a reported 90-day survival of 45% [14]. Risk factors associated with poor outcomes include persistent neutropenia and the use of corticosteroids [27]. Few longitudinal studies have examined the trends in presentation and treatment outcomes [12,14]. Historically, treatment outcomes have been evaluated using mortality rates at fixed intervals (e.g., 6 weeks and 12 weeks) with limited information about the specific response to antifungal treatment. In 2008, the European Organisation for Research and Treatment of Cancer and Mycoses Study Group Education and Research Consortium (EORTC/MSG), based on clinical trial definition, considered patients who die while receiving antifungal therapy as having failed treatment [28]. However, this view might be dated/inadequate considering advancements in the management of invasive fungal infections and treatment of HMs [29]. In the real-world context, deaths may be attributable to underlying malignant disease or other comorbidities rather than the fungal infection.

In this study, we evaluated 22 cases of invasive fusariosis over 14 years at a Singaporean tertiary medical centre. We examined each patient’s clinical course and outcome and aimed to determine a “true” antifungal treatment response based on clinical, microbiological, and radiological criteria.

## 2. Materials and Methods

### 2.1. Clinical Data Collection

This is a retrospective single centre study of adult patients with invasive *Fusarium* infections treated at the Singapore General Hospital (SGH), the largest acute tertiary referral centre (with established Haematology, Oncology, Transplant services and home to the regional Burns Unit in Southeast Asia) from January 2010 to December 2023.

Cases of invasive *Fusarium* infections were identified by retrospective surveillance of all positive fungal culture results from any site as part of the SGH Fungal Registry, approved by the SingHealth Institutional Review Board (CIRB: 2021/2293, year 2021). There were 78 *Fusarium* isolates identified. After excluding 56 superficial infections, 22 cases of probable and proven invasive *Fusarium* infections based on the 2020 EORTC/MSG criteria [30] were included in the study.

Patient’s baseline demographics, clinical, microbiological and radiological features pertaining to the Fusarium infection were collected. Treatment and clinical outcomes were also evaluated. The data was captured in the Redcap electronic data system and subsequently analysed.

### 2.2. Microbiological Diagnosis and Anti-Fungal Susceptibility Testing

*Fusarium* species is identified phenotypically by the typical canoe-shaped conidia under general microscopy and confirmed genotypically by internal transcribed sequencing.

Antifungal susceptibility testing, including voriconazole, posaconazole, isavuconazole and amphotericin B, was carried out on request by the managing Infectious Diseases (ID) physician. Voriconazole, posaconazole and amphotericin B MICs were carried out using gradient diffusion strips (bioMérieux; Marcy-l’Etoile, France) from 2011 and Sensititre Yeast One assay (YO10) by Trek Diagnostics Systems (ThermoFisher Scientific; West Sussex, United Kingdom) from 2016 onwards. Isavuconazole was performed using gradient diffusion strips (Liofilchem; Roseto degli Abruzzi, Italy). All gradient diffusion strips were placed on RPMI 1640 plates supplemented with 2% glucose and incubated at 35 °C. These methods are carried out according to the manufacturers’ instructions.

### 2.3. Determination of Outcomes

The outcome of each episode of *Fusarium* infection was evaluated based on clinical, microbiological, and radiological attributes. Mortality was determined at the last contact, defined as the date of the end of antifungal treatment assessment or death, whichever occurred first. Where death occurred, two independent ID physicians determined whether it was directly, indirectly, or not attributable to *Fusarium* infection. When discrepancies arose between their assessments, the opinion of a third ID physician was sought to reach a consensus. Additionally, all patients with HM and invasive fungal infections were co-managed with Haematologists. Management decisions and causes of death were discussed during clinical rounds and documented.

## 3. Results

### 3.1. Patient Characteristics and Risk Factors for Invasive Fusariosis

There were 22 patients with invasive fusariosis, and their demographics are summarised in Table 1. Majority of the patients, 17/22 (77%) patients had an underlying diagnosis of an HM, of which, acute leukemia was the most common (76.5%), followed by myelodysplastic syndrome (11.8%), aplastic anaemia (5.9%) and lymphoma (5.9%). Of these HM patients, 5/17 (29.4%) received ASCT; 1 patient had allograft failure and pancytopenia post SCT, another was transplanted in the past month, and 3 had relapsed HM after ASCT. Of note, 15/17 (88.2%) patients with HM were on antifungal prophylaxis as detailed in Table 1. None were on voriconazole prophylaxis.

Apart from HM, the remaining five patients who developed invasive fusariosis had other risk factors; 2 (9.1%) had extensive burns, 1 (4.5%) with newly diagnosed Acquired Immunodeficiency Syndrome and Pneumocystis Jerovecii Pneumonia, 1 (4.5%) patient had a history of short gut syndrome on lifelong total parenteral nutrition and prolonged central vascular catheter use and the last patient was an elderly male with acute respiratory distress syndrome requiring high dose corticosteroids.

At the time of diagnosis of invasive fusariosis, most subjects, 21/22 (95.5%) were on immunosuppressive treatment; 17/22 (77.3%) were on chemotherapy, 9/22 (40.9%) were on corticosteroids, and 13/22 (59.1%) were on novel therapeutics for HM.

### 3.2. Clinical Characteristics of Invasive Fusariosis

Based on the EORTC/MSG criteria, there were 16 (72.7%) and 6 (27.3%) cases of proven and probable invasive fusariosis respectively. Table 2 provides detailed patient case descriptions and clinical outcomes.

The infection was disseminated in 13 (59.1%) cases. Pulmonary infections were most common, and the typical radiological findings include dense, well-circumscribed lesions and consolidation. Sinus involvement was also common (22.7% of cases), and all had opacification of the sinuses on imaging. Among those with disseminated infection, all had cutaneous lesions, 2 patients had central nervous system lesions, and 1 patient had hepatosplenic involvement.

### 3.3. Characteristics of Fusarium Isolated and Antifungal Treatment

Eighteen isolates had species complex identification, 16/18 (88.9%) were *Fusarium solani* species complex and 2/18 (11.1%) were *Fusarium oxysporum* species complex. Four isolates did not have further speciation done.

MIC testing for antifungals was conducted at the request of the managing physician, with the results presented in Table 3. Susceptibility testing for 2 of the isolates collected between the years 2011–2016 was performed using gradient diffusion strips (bioMérieux; Marcy-l’Etoile, France), and susceptibility testing of the remaining 11 isolates was performed using the Sensititre Yeast One assay (YO10) by Trek Diagnostics Systems (ThermoFisher Scientific; West Sussex, United Kingdom). Isavuconazole susceptibility testing was performed using gradient diffusion strips (Liofilchem; Roseto degli Abruzzi, Italy). A general trend of lower MICs for voriconazole and amphotericin B was observed, while posaconazole MICs were higher. A range of MICs was observed with isavuconazole. However, the sample size was not sufficiently large to draw any conclusions.

Antifungal treatment used is detailed in Table 4. 21 patients received anti-fungal therapy and 1 patient received palliative treatment. Combination antifungal treatment was used at diagnosis in 12/21 (57.1%) and liposomal amphotericin B with voriconazole was the most used combination. The remaining 9 (42.9%) patients were started on monotherapy, either liposomal amphotericin B or voriconazole. Among those started on combination antifungal therapy, 7 patients were transitioned to monotherapy with improvement in their infections, while 5 patients remained on combination therapy because of challenges in controlling the infection. Voriconazole was mostly frequently used as stepdown monotherapy (7/21, 33.3%). The median duration of treatment was 53 (IQR 17.5–103.5) days. Nine out of 21 (42.9%) patients experienced adverse events; 5 (23.8%) developed nephrotoxicity due to liposomal amphotericin B, 3 (14.3%) experienced triazole associated hepatotoxicity and 1 (4.8%) patient on voriconazole developed altered mental status.

Other adjunctive treatments include surgery, the use of granulocyte stimulating factor, reduction of immunosuppression, and removal of the central venous catheter. See Table 4 for more details.

### 3.4. Outcomes and Mortality

The evaluable population comprised twenty patients, as two patients were transferred overseas and their final outcome was unknown.

After thoroughly evaluating each patient’s response to antifungal treatment, we found that 10 of 20 patients (50%) achieved resolution of invasive *Fusarium* infection and were alive at the last contact. The remaining 50% of patients died, with 9/10 (90%) deaths occurring within 3 months and 1 (10%) death occurring within 6 months from the diagnosis. Only 2 deaths, were directly attributable to *Fusarium* infection. Details of the mortality outcomes are presented in Table 5.

Due to the small number of patients in this case series, it was not feasible to conduct a statistical analysis. Nonetheless, there appeared to be a trend indicating higher mortality rates among patients with prolonged neutropenia, disseminated infection, and those receiving corticosteroids. The mortality rate of patients with disseminated infection is high, 10/13 (76.9%); of which half died < 3 months from the diagnosis of *Fusarium* infection. 5/8 (62.5%) patients with prolonged neutropenia and 3/6 (50%) of patients on corticosteroids died before the end of treatment visit.

## 4. Discussion

In this study, we report the detailed clinical and microbiological features and outcomes of invasive *Fusarium* infections over a 14-year period at a tertiary hospital in Singapore. To the best of our knowledge, this is a large case series of *Fusarium* infection from a tertiary hospital in Asia reporting on both survival outcomes as well as nuanced response to treatment based on clinical, microbiological and radiological parameters. Other reported Asian case series include those from South Korea [10] and India [11,17].

The experience at our centre is similar to that reported by other centres internationally. The majority of the cases involved immunocompromised patients, particularly those with HM. The most affected sites include the lungs or skin, and fungaemia was observed in approximately a third of the cases in our series [1,2,31].

In our study, mortality was 50%, comparable with global data, ranging from 56% to 59% in Europe [32,33], 50–57% in the Americas [14,31] and 50–60% in Asia [10,11]. Similarly, in our cohort, mortality was the highest in patients with disseminated infection, prolonged neutropenia and those receiving corticosteroid treatment [27,34].

Based on the 2008 EORTC/MSG criteria for defining responses to antifungal therapy [28], patients who die while receiving antifungal therapy would be considered to have failed antifungal therapy, regardless of the cause.

However, using death as a key determinant for treatment failure may be oversimplifying a complicated disease process. We stand with proponents calling for a revision of this criteria [29] and advocate for a more wholistic approach taking into consideration clinical, microbiological and radiological parameters. By published definitions, 50% of our patients would have failed antifungal treatment. Yet, on detailed chart review, only 2 deaths were directly attributable to *Fusarium* infection, and the other patients died from causes where fusariosis was not directly contributory, such as extensive burns and complications, severe bacterial pneumonia, viral pneumonia, polymicrobial bacteraemia and hepatic abscesses and relapsed HM. Accurate and precise definitions of treatment response, best reflecting clinical practice, is crucial for trial design and to inform treatment strategies for challenging infections like Fusariosis. An objective and simple definition for treatment response or success is useful but may misinform clinicians regarding the true efficacy of anti-fungal therapy for *Fusarium* infections [28,29], with the unintended consequences of premature termination of antifungal treatment in the worst-case scenario. For best outcomes, immune recovery, treatment of co-infections, and treatment of underlying disease such as the HM, is important.

A key observation in our series was that the time to definitive treatment from the onset of symptoms was typically less than a day. This is most likely due to seasoned clinicians who have developed strong collaborative relationships between multidisciplinary teams, thereby facilitating timely diagnosis, and treatment. Also, over half of the patients were started on combination antifungal therapy at presentation. Whether the above factors contributed directly to the reasonably high rate of infection resolution (~50%) or not, a collaborative and multi-disciplinary approach to the management of *Fusarium* infection is beneficial.

Our study had several limitations. Firstly, it is a single-centre study with centre-specific practices. Hence, our results may not be generalisable. Secondly, due to the rarity of invasive *Fusarium* infections, this is a small study, and statistical analyses could not be performed on the factors influencing treatment outcomes. Thirdly, the selection of antifungal treatment in our study was physician-dependent, and our data may reflect local prescribing patterns of treating physicians rather than inform on the efficacy of therapy. Lastly, adjudication of cases were carried out solely by ID physicians which may under or overestimate the contributory effect of invasive fusariosis on mortality. Moreover, due to the complexity of the cases, we acknowledge that it is challenging to determine attributable mortality due to fusariosis for all cases. A mitigating factor would be that all cases of patients with HM and invasive fusariosis were co-managed with haematologists, and causes of death were discussed and detailed on the charts.

In summary, we present a tertiary centre’s 14 years of experience with invasive *Fusarium* infections in Singapore. The high mortality rates may not be directly attributable to *Fusarium* infections but instead be related to factors beyond the infection itself. We believe that it may be time to challenge the status quo and redefine how we assess treatment outcomes for invasive mould infections. Nonetheless, invasive *Fusariosis* remains a serious infection affecting the most immunocompromised patients and has high mortality rates. Indeed, with newer approaches to treating malignant diseases, there is an ever-increasing number of immunocompromised patients at risk for invasive fungal infections [35]. It is imperative that we continue our best practices and look to the horizon for newer treatment approaches [28] that would ultimately benefit our patients.

## Figures and Tables

**Table 1:** Baseline demographics of patients with invasive *Fusarium* infection.

Characteristic	No. (%)
Age, median (IQR), years	58 (52.3, 66.5)
Male sex	13 (59.1)
Race and ethnicity	
Chinese	16 (72.7)
Indian	3 (13.6)
Malay	1 (4.5)
Others	2 (9.1)
Haematologic malignancy	17 (77.3)
Acute myeloid leukemia	9 (40.9)
Acute lymphoid leukemia	2 (9.1)
Acute undifferentiated leukemia	2 (9.1)
Myelodysplastic syndrome	2 (9.1)
Lymphoma	1 (4.5)
Aplastic anaemia	1 (4.5)
Relapsed refractory haematologic malignancy	8 (36.4)
Neutropenia	12 (54.5)
Prior allogenic stem cell transplant ^1^	5 (22.7)
Antifungal prophylaxis	
Itraconazole	6 (27.3)
Posaconazole	4 (18.2)
Fluconazole	1 (4.5)
Caspofungin	5 (22.7)
Other conditions predisposing to invasive *Fusarium* infection	
Severe burns	2 (9.1)
AIDS	1 (4.5)
Central venous catheter	1 (4.5)
Severe pneumonia and ARDS	1 (4.5)
Use of corticosteroids	9 (40.9)
Use of novel therapeutics	
Venetoclax	4 (18.2)
Gilteritinib	3 (13.6)
Sorafenib	2 (9.1)
Midostaurin	2 (9.1)
Inotuzumab	1 (4.5)
Ponatinib	1 (4.5)

^1^ Four patients had acute myeloid leukemia, one patient had myelodysplastic syndrome. Abbreviation: IQR, Interquartile range.

**Table 2:** Detailed case description of patients with invasive *Fusarium* infection, treatment and outcomes.

Age/Sex	Underlying Disease	Diagnosis *	Sites of Infection	Species of *Fusarium*	Therapy at Diagnosis	Subsequent Therapy	Treatment Duration (Days)	Response to Treatment	Outcome
65/M	Advanced Immunodeficiency Disease Syndrome, Pneumocystis Carinii Pneumonia	Probable	Lung	*Fusarium solani*	Liposomal amphotericin B	Liposomal amphotericin B	14	Improvement clinically and radiologically	Death from severe nosocomial pneumonia
60/F	Newly diagnosed acute undifferentiated leukaemia on chemotherapy	Proven	Eye, skin	*Fusarium solani*	Liposomal amphotericin B and Posaconazole	Posaconazole	Unknown	Skin lesions improved; visual prognosis uncertain	Care transferred to Cambodia
63/F	Acute undifferentiated leukaemia, on chemotherapy	Probable	Lung	*Fusarium solani*	Voriconazole and liposomal amphotericin B	Posaconazole and liposomal amphotericin B	Unknown	Improvement radiologically	Care transferred to India
55/M	Newly diagnosed acute myeloid leukaemia, failure of induction chemotherapy, prolonged neutropenia	Probable	Lung	*Fusarium solani*	Liposomal amphotericin B	Posaconazole, later switched to liposomal amphotericin B	90	Initial improvement clinically and radiologically	Death from HM disease progression, superimposed bacterial pneumonia
54/M	Acute myeloid leukaemia, twice ASCT with allograft failure and persistent pancytopenia	Probable	Sinuses, facial cellulitis	*Fusarium* species	Posaconazole	Posaconazole	198	Infection progressed at time of death	Death from fusariosis
47/M	Extensive burns, septic shock from polymicrobial bacteraemia and receipt of corticosteroids	Proven	Skin and soft tissue, fungaemia	*Fusarium* species	Liposomal amphotericin B	Liposomal amphotericin B	50	Clearance of *Fusarium* from blood cultures	Death from burns and complications
73/M	Newly diagnosed acute myeloid leukaemia on chemotherapy	Probable	Lung	*Fusarium* species	Voriconazole	Voriconazole	8	Unable to tell, short duration of illness before death	Death from Metapneumovirus influenza and *S maltophila* pneumonia
41/F	Relapsed acute myeloid leukaemia, received ASCT in remission	Proven	Sinuses, skin, bilateral eyes, fungaemia	*Fusarium solani* species complex	Voriconazole and liposomal amphotericin B	Voriconazole	325	Clinical resolution	Resolved
68/F	Refractory peripheral T cell lymphoma, ongoing chemotherapy, corticosteroids, prolonged neutropenia	Proven	Sinuses, eyes, skin, fungaemia	*Fusarium solani* species complex	Voriconazole and liposomal amphotericin B	Liposomal amphotericin B	36	Clearance of *Fusarium* from blood cultures	Death from lymphoma
52/F	Acute lymphoblastic leukaemia, on chemotherapy, prolonged neutropenia	Proven	Skin and muscles of foot, fungaemia	*Fusarium solani* species complex	Voriconazole and liposomal amphotericin B	Posaconazole and liposomal amphotericin B	60	Clinical improvement and clearance of *Fusarium* from blood cultures	Resolved
53/M	Short gut syndrome on lifelong total parenteral nutrition	Proven	Catheter related bloodstream infection	*Fusarium oxysporum* species complex	Liposomal amphotericin B	Liposomal amphotericin B	21	Clinical improvement and clearance of *Fusarium* from blood cultures	Resolved
57/F	Chronic myeloid leukaemia in lymphoid blast crisis on treatment with corticosteroid use	Proven	Paronychia of toe, lung	*Fusarium solani* species complex	Voriconazole and liposomal amphotericin B	Liposomal amphotericin B	53	Clinical and radiological improvement	Resolved
73/F	Myelodysplastic syndrome with prolonged neutropenia	Proven	Fungaemia	*Fusarium solani* species complex	Not treated, for palliative management		NA	Unable to access	Death from polymicrobial bacteraemia, cholangitis and hepatic abscesses
22/M	Refractory acute myeloid leukaemia	Proven	Skin and lung	*Fusarium oxysporum* species complex	Voriconazole and liposomal amphotericin B	Voriconazole	117	Clinical and radiological resolution	Resolved
56/M	Relapsed myelodysplastic syndrome, received ASCT, on chemotherapy	Proven	Paronychia of toe, skin, submandibular abscess, lung, brain	*Fusarium solani* species complex	Voriconazole and liposomal amphotericin B	Isavuconazole and liposomal amphotericin B	37	Mixed response: skin lesions improved, lung lesions progressed	Death from relapsed MDS
67/M	Relapsed acute myeloid leukaemia on chemotherapy with prolonged neutropenia	Proven	Right calf abscesses, osteomyelitis of toe, paronychia, lung	*Fusarium solani* species complex	Isavuconazole	Voriconazole	59	Clinical and radiological improvement	Resolved
75/F	Refractory aplastic anaemia with prolonged neutropenia	Proven	Skin, lung, fungaemia	*Fusarium solani* species complex	Voriconazole and liposomal amphotericin B	Voriconazole and liposomal amphotericin B	6	Fever lysed	Death from fusariosis
65/F	Relapsed acute myeloid leukaemia, received ASCT and prolonged neutropenia	Proven	Paronychia of thumb, nodules, liver, spleen, intramuscular lesions, osteomyelitis, chorioretinal abscess	*Fusarium solani* species complex	Voriconazole and liposomal amphotericin B	Voriconazole and liposomal amphotericin B	325	Clinical and radiological improvement	Resolved
31/M	Extensive burns, septic shock, on corticosteroids	Proven	Soft tissue, eye, fungaemia	*Fusarium solani* species complex	Voriconazole and liposomal amphotericin B	Liposomal amphotericin B	11	Clearance of *Fusarium* from blood cultures	Death from burns and complications
83/M	Severe community acquired pneumonia, septic shock, Acute Respiratory Distress syndrome	Probable	Lung	*Fusarium* species	Voriconazole	Voriconazole	14	Clinical stability, no regrowth from sputum culture	Resolved
59/M	Acute myeloid leukaemia, received ASCT	Proven	Skin, sinusitis	*Fusarium solani* species complex	Voriconazole and liposomal amphotericin B	Voriconazole	156	Clinical improvement	Resolved
47/F	Newly diagnosed acute myeloid leukaemia on induction chemotherapy	Proven	Sinus	*Fusarium solani* species complex	Liposomal amphotericin B	Voriconazole	61	Clinical and radiological improvement	Resolved

* Based on the 2020 Revision and Update of the Consensus Definitions of Invasive Fungal Disease From the European Organization for Research and Treatment of Cancer and the Mycoses Study Group Education and Research Consortium [31].

**Table 3:** Results of antifungal susceptibility testing of the isolates.

	MICs of Antifungal Agents Against 13 Clinical Isolates of *Fusarium solani* Species Complex or *Fusarium oxysporum* Species Complex (ug/mL)										
	Voriconazole				Posaconazole	Isavuconazole			Amphotericin B		
	2	4	8	>8	>8	0.25	1	>32	2	4	8
*Fusarium solani* species complex (11)	1		3	7	10		1	4	4	5	2
*Fusarium oxysporum* species complex (2)		1	1		2	1				2	

**Table 4:** Treatment of *Fusarium* infections.

Characteristic	No. (%)
Anti-fungal treatment	
Monotherapy	9 (40.9)
Combination therapy	12 (54.5)
No treatment	1 (4.5) ^1^
Monotherapy at the start	9 (40.9)
Type of monotherapy	
Voriconazole	2 (9.1)
Posaconazole	1 (4.5)
Isavuconazole	1 (4.5)
Amphotericin B	5 (22.7)
Combination therapy at start	12 (54.5)
Type of combination therapy	
Liposomal Amphotericin B and Voriconazole	11 (50)
Liposomal Amphotericin B and Posaconazole	1 (4.5)
Final treatment regimen	
Posaconazole	2 (9.1)
Voriconazole	7 (31.8)
Amphotericin B	6 (27.3)
Posaconazole and Amphotericin B	3 (13.6)
Voriconazole and Amphotericin B	2 (9.1)
Isavuconazole and Amphotericin B	1 (4.5)
Treatment, median (IQR) days	53 (17.5–103.5)
Adjunctive measures	
Surgery	9 (40.9)
Burns debridement	2 (9.1)
Aspiration of superficial abscess	2 (9.1)
Functional Endoscopic Sinus Surgery	3 (13.6)
Vitrectomy	1 (4.5)
Evisceration of eye	1 (4.5)
Reduction of immunosuppression	5 (22.7)
G-CSF administered	6 (27.3)
Exchange of central venous catheter	1 (4.5)

^1^ Patient received palliative care.

**Table 5:** Outcomes of invasive Fusarium infection in the evaluable population.

Outcome	No. (%)
Fusariosis resolved and alive at last contact	10 (50)
Time to resolution, IQR, days	54, 17.5–103.5
Total number of deaths	10 (50)
Death < 3 months of diagnosis of invasive fusariosis	9 (45)
Death directed related to invasive fusariosis	1 (5)
Death contributed by but not directly caused by fusariosis	8 (40)
Death < 6 months of diagnosis of invasive fusariosis	1 (5)
Death directed related to invasive fusariosis	1 (5)
Death contributed by but not directly caused by fusariosis	0
Death > 1 year of diagnosis of invasive fusariosis	0

Reasons for death: Two from extensive burns and complications, one from severe nosocomial pneumonia, one from disease progression and bacterial pneumonia, one from influenza metapneumovirus and *S.maltophila* pneumonia, one from lymphoma, one from polymicrobial bacteraemia and hepatic abscesses, one from relapsed myelodysplastic syndrome.

## Data Availability

The original contributions presented in this study are included in the article. Further inquiries can be directed to the corresponding author(s).
